# Effect of a 2-hour interval between dinner and bedtime on glycated haemoglobin levels in middle-aged and elderly Japanese people: a longitudinal analysis of 3-year health check-up data

**DOI:** 10.1136/bmjnph-2018-000011

**Published:** 2019-01-21

**Authors:** Su Su Maw, Chiyori Haga

**Affiliations:** Community Health Nursing, Graduate School of Health Sciences, Okayama University, Okayama City, Japan

**Keywords:** dietary patterns, diabetes mellitus, metabolic syndrome, lifestyle factors

## Abstract

**Introduction:**

There is a need for evidence-based measures to examine the risk factors for lifestyle-related diseases. In Japan, a 2-hour interval between dinner and sleep is recommended as a healthy practice. However, the effect of an appropriate duration between dinner and bedtime on glycated haemoglobin (HbA1c) levels remains unclear. This study aimed to identify the effect of a duration of 2 hours or shorter between dinner and bedtime on HbA1c levels in middle-aged and elderly Japanese individuals.

**Methods:**

A longitudinal analysis of health check-up data (2012, 2013 and 2014) was performed. Lifestyle and anthropometric data of individuals aged 40–74 years who did not have any pre-diabetic and diabetic conditions were collected for multilevel analysis. Univariate analysis was performed to assess the influence of each lifestyle variable. Then, two-level random intercept models were created using statistical software SAS 9.3 (SAS Institute Inc, Cary, NC, USA).

**Results:**

The cohort comprised 1573 individuals in 2012, two-thirds of whom were women. The mean HbA1c level was 5.20% in 2012 and 5.58% in 2013 and 2014. A total of 83 (16.1%) men and 70 (7.5%) women fell asleep within 2 hours after dinner. The influence of ensuring a 2-hour interval between dinner and bedtime did not have a remarkable effect on increasing HbA1c levels. The regression coefficient of 2-hour interval and HbA1c levels over time was −0.02 (p=0.45). Smoking (p=0.013), alcohol consumption (p=0.010) and higher body mass index (BMI) (p<0.001) may have influenced HbA1c trends.

**Conclusion:**

Durations of 2 hours or shorter between dinner and bedtime did not influence HbA1c changes in middle-aged and elderly Japanese people. Instead, the focus should be on maintaining a normal BMI and abstaining from smoking and consuming alcohol to ensure stable HbA1c patterns in the long term.

## Introduction

An annual increase has been observed in the incidence rates of several lifestyle-related diseases including obesity, metabolic syndrome (MetS) and cardiovascular disease, primarily due to people’s unhealthy lifestyle habits.^1^ As such, healthcare policies that include lifestyle assessments and interventions should be implemented to combat the complications associated with unhealthy lifestyles.^2 3^ Increasing evidence demonstrates the effectiveness of lifestyle modifications in reducing the risk of developing lifestyle-related diseases.^4^ The use of evidence-based practice (EBP) is required to effectively prevent lifestyle-related disease development. One EBP principle is to provide the scientific rationale for interventions.^5^ Like in other developed nations, lifestyle-related diseases are also prevalent in Japan, and the Japanese government has implemented a national health promotion programme to address the issues associated with various non-communicable diseases.^6^ Although traditional Japanese food and eating styles are healthy, owing to large variety, small portion sizes, inclusion of soups, high vegetable intake and high water content,^7^ Westernisation has led to an increase in the proportion of people with unhealthy dietary habits.^8 9^ As a result, there is a steady increase in people’s calorie intakes, as well as the popularity of fast foods and beverages.^8^

Given that the prevalence of lifestyle-related diseases increases with age,^10^ the Japanese government pays particular attention to elderly people in the prevention of these diseases. The ageing population in Japan is growing substantially and 27.3% of the country’s population are aged ≥65 years.^11^ A new screening and intervention programme called ‘Specific health check-ups and specific health guidance’, specially designed to address lifestyle-related diseases, was launched in 2008.^12^ As part of this, the lifestyle habits and biophysical characteristics of individuals aged 40–74 years are assessed using the structured questionnaire developed by the Japanese Ministry of Health, Labour and Welfare. One of the factors assessed in terms of eating habits is ensuring a 2-hour interval between dinner and bedtime more than three times a week, as it is assumed that a shorter interval could be a risk factor for the development of lifestyle-related diseases.^13^ Short sleep durations and sleep deprivation are related to unhealthy eating habits and impaired glucose metabolism.^14–16^ Altered sleep duration can lead to obesity-related morbidities. Animal and human experimental models have shown the impact of circadian misalignment on metabolic factors that lead to the development of metabolic diseases such as hyperglycaemia and hyperlipidaemia.^17^ Glycated haemoglobin (HbA1c) is a biomarker for the presence and severity of hyperglycaemia, and it reflects the presence of diabetes or pre-diabetes over time.^18^ Higher HbA1c levels may also be predictive of an increased risk for cardiovascular disease mortality.^19^ HbA1c measurement is included in the Japanese health examination programme for the identification of diabetes.^13^

The Japanese government aims to decrease the percentage of individuals with elevated blood glucose levels (HbA1c ≥8.4%) to 1.0% in 2022.^20^ Some factors that contribute to increased HbA1c levels include the consumption of dinner late at night^21^ and short or long sleep durations^22^; in contrast, skipping breakfast and eating speed are not associated with HbA1c.^21 23^ However, faster eating rates were found to be associated with abnormal glucose metabolism and obesity development.^24^ People consume the largest amount of food late at night compared with the other times of day.^25^ The public health programme recommends participation in physical activity and the consumption of a healthy diet to reduce the risk of obesity.^4^ However, the effect of ensuring a 2-hour interval between dinner and sleep on HbA1c levels is currently unclear. There is no clear evidence supporting the appropriateness of ensuring such a 2-hour interval, and the corresponding effects on the human metabolic status are controversial. Thus, this study aimed to identify the effect of a duration of 2 hours or shorter between dinner and bedtime on HbA1c levels in middle-aged and elderly Japanese people with no underlying diabetes-related conditions over a 3-year period.

## Methods

### Study design

In this observational cohort study, we used health check-up data obtained in 2012 for the participants’ baseline health status (Dryad identifier doi: 10.5061/dryad.kg183m5). Lifestyle habits such as ensuring a duration of 2 hours between dinner and bedtime and HbA1c levels were followed-up for 2 years to determine the effect of lifestyle habits on HbA1c changes.

### Study population and enrolment

In this study, we analysed data on individuals who underwent a specific health examination in City A in Okayama Prefecture, Japan, from 1 April 2012 to 31 March 2014. Health examinations for self-employed or individuals aged ≥40 years are mandatory in Japan. The purpose of this specific health examination is to identify and prevent MetS in adults aged 40 to 74 years. Therefore, the participants in this study were adults aged 40 to 74 years, who were self-employed or retired.

In Japan, the cost of health check-ups is covered by the individual's insurance company, and the coverage ranges from 30% to full subsidy. Medical insurance companies aim to reduce the medical costs associated with lifestyle-related diseases as they account for 60% of all deaths in Japan. They analyse health check-up data to assess health conditions and develop measures for disease prevention and health promotion. The participants in this study paid only 1000 Japanese Yen (£7, €8) and were free to choose their preferred medical institution for the health check-up.

All data were anonymised and maintained in a state of no concatenation. Given the observational design of this study and the use of existing data, informed consent was not obtained from individual participants.^26^ This study was approved by the Ethical Review Board of Okayama University Graduate School of Medicine, Dentistry and Pharmaceutical Sciences and Okayama University Hospital (reference number 1605-015-001).

### Lifestyle and anthropometric information

During the health examinations, participants were asked to answer a 22-item self-report questionnaire regarding their lifestyle habits. The questionnaire included questions on smoking history (≥100 cigarettes in the past year), participation in regular exercise (at least 30 min, three times a week), weight gain of over 10 kg since age 20 years, weight gain ≥3 kg within the last year, fast and slow eating style, intake of alcohol nearly every day, skipping breakfast, and sleeping within 2 hours after dinner three times a week. Responses were categorised as ‘yes’ or ‘no’ to promote understanding and improve the answering rate.

Body weight and height were measured by registered healthcare professionals via standardised methods. Height was measured in units of millimetres and recorded in centimetres. Weight was measured using digital scales in kilogrammes with the participant wearing light clothing and no shoes. Body mass index (BMI; kg/m^2^) was calculated from the measured weight and height. A BMI of 25 kg/m^2^ was regarded as the cut-off point for obesity, as proposed by the Japan Society for the Study of Obesity.^27^ In addition, triglyceride (TG) and blood pressure (BP) values were also measured to determine the risks of MetS development by considering the multicollinearity between them and the main predictor variable.

The fasting blood glucose, HbA1c, high-density lipoprotein, low-density lipoprotein, glutamic oxaloacetic transaminase, glutamic pyruvic transaminase, γ-glutamyl transpeptidase, serum creatinine, haematocrit and haemoglobin values, and the estimated glomerular filtration rate were measured. All data were converted and analysed by specialist clinical laboratory technicians.

### Statistical analysis

Participants’ baseline lifestyle characteristics and anthropometric measures were assessed according to sex. χ^2^ tests were used for categorical variables, while Student’s t-tests and Mann-Whitney U tests were used for continuous variables. We examined the effect of a 2-hour interval between dinner and bedtime in increasing HbA1c levels and other lifestyle and anthropometric variables using univariate analyses. Then, we used a two-level random intercept model as follows: the time of measurement was set to level 1, and the individual variables were set to level 2. We used BMI, TG and BP, which had significant relationships with HbA1c, and smoking, alcohol consumption and regular participation in exercise, which may be associated with the outcome, as covariates. Regression coefficients of those variables were assessed by developing statistical models. For level 1, we specified the BMI measurement timing (in the first, second or third year) as ordinal. For level 2, we used the fixed effects of the interval between dinner and bedtime, smoking, regular exercise, TG, BP and BMI at the baseline. All statistical analyses were conducted with the statistical software SAS 9.3 (SAS Institute Inc, Cary, NC, USA) using the MIXED procedure for multilevel analyses with maximum likelihood estimation. To evaluate the association between the level 2 predictor variables and the HbA1c trajectory, we modelled these individual variables and the interaction terms for each individual variable as well as the time variable.

### Patient and public involvement

Community residents or patients were not included in the elaboration of the research questions and study design. The results of this research will be distributed to stakeholders such as the health centre of City A, Okayama prefecture, Japan, and Okayama University, Japan, after publication in a scientific journal.

## Results

### Participant characteristics

Of the eligible participants, those with abnormal HbA1c levels (≥6.1%) were excluded to prevent the influence of pre-diabetes and diabetes-related conditions at the baseline. A total of 1573 people (579 men and 994 women) were initially included in this study. Of these, 99 (5.9%) had high blood glucose levels. Finally, 1531 individuals (561 men and 970 women) who underwent health check-ups for at least 2 years from 2012 to 2014 were included. Table 1 shows the distributions of the key variables. Most of the variables were different between the men and women. Women were healthier, with lower BMI (p<0.001), systolic BP (p=0.038) and TG (p<0.001) values than men. They also had a healthier lifestyle with a decreasing smoking status (p<0.001) and lower alcohol consumption (p<0.001). However, there was no significant difference in the age (p=0.72), mean HbA1c level (p=0.78) and exercise participation pattern (p=0.939). The number of women who slept within 2 hours after dinner was significantly lower than that of men (70 (7.5%) vs 83 (16.1%); p<0.001).

**Table 1:** Characteristics of participants at baseline (year 2012)

Variables	Men						Women						P value
	Number of participants		Number of missing		Mean/no.	SD/%	Number of participants		Number of missing		Mean/no.	SD/%	
	n	%	n	%			n	%	n	%			
Demographic data													
Age (years)*	579	100			65.0	8.4	994	100			64.8	7.6	0.72
Body mass index*	579	100			23.5	3.1	994	100			22.5	3.4	<0.001
Systolic BP (mmHg)*	579	100			131.4	17.9	994	100			129.4	17.7	0.038
Diastolic BP (mmHg)*	579	100			77.2	11.0	994	100			75.0	10.9	<0.001
Blood examination													
HbA1c (%)*	579	100			5.2	0.3	994	100			5.2	0.3	0.78
TG (mg/dL)†	579	100			132.2	80.0	994	100			105.3	52.0	<0.001
HDL (mg/dL)†	579	100			57.5	16.2	994	100			65.0	15.4	<0.001
LDL (mg/dL)†	579	100			120.6	30.1	994	100			130.8	31.9	<0.001
GOT (U/L)†	579	100			25.1	10.5	994	100			22.6	8.8	<0.001
GPT (U/L)†	579	100			23.9	13.6	994	100			19.0	11.6	<0.001
γ-GTP (U/L)†	579	100			50.9	56.2	994	100			24.1	27.2	<0.001
Lifestyle													
Short duration between dinner and bedtime (Yes)‡	516	89.1	63	10.9	83	16.1	928	93.4	66	6.6	70	7.5	<0.001
Smoking (Yes)‡	579	100.0			124	21.4	994	100			24	2.4	<0.001
Weight gain >3 kg within 1 year (Yes)‡	515	88.9	64	11.1	112	21.7	927	93.3	67	6.7	156	16.8	0.024
Consuming snacks after dinner (Yes)‡	516	89.1	63	10.9	51	9.9	928	93.4	66	6.6	112	12.1	0.225
Skipping breakfast (Yes)‡	516	89.1	63	10.9	38	7.4	928	93.4	66	6.6	26	2.8	<0.001
Drinking alcohol (Yes)‡	516	89.1	63	10.9	264	51.2	928	93.4	66	6.6	54	5.8	<0.001
Regular exercise (Yes)‡	579	100.0	16	2.8	264	45.6	994	100	43	4.3	444	44.7	0.939

*P values were calculated with a Student's t-test.

†P values were calculated with a Mann-Whitney U test.

‡P values were χ^2^ test with Yates.

BP, blood pressure; GOT, glutamicoxaloacetic transaminase; GPT, glutamic pyruvic transaminase; GTP, glutamyltranspeptidase; HDL, high-density lipoprotein; HbA1c, glycated haemoglobin; LDL, low-density lipoprotein; TG, trigyceride.

Table 2 shows the HbA1c distribution at each time-point. The mean±SD value of HbA1c was 5.20±0.34 in 2012; it increased to 5.58±0.35 in 2013, and was maintained at 5.58 in 2014. There was no remarkable difference in the slope of this trajectory between men and women. However, this trajectory could be uncertain as we did not have all the data pertaining to the three time-points.

**Table 2:** Number of participants and glycated haemoglobin (HbA1c) ranges

Time point of measurement	All		Men		Women	
	n	Mean±SD	n	Mean±SD	n	Mean±SD
2012	1573	5.20±0.31	579	5.20±0.34	994	5.20±0.30
2013	1190	5.58±0.35	416	5.56±0.40	774	5.60±0.31
2014	1032	5.58±0.43	356	5.55±0.42	676	5.59±0.43

### Changes in HbA1c levels associated with falling asleep within 2 hours after dinner

**Figure 1:**
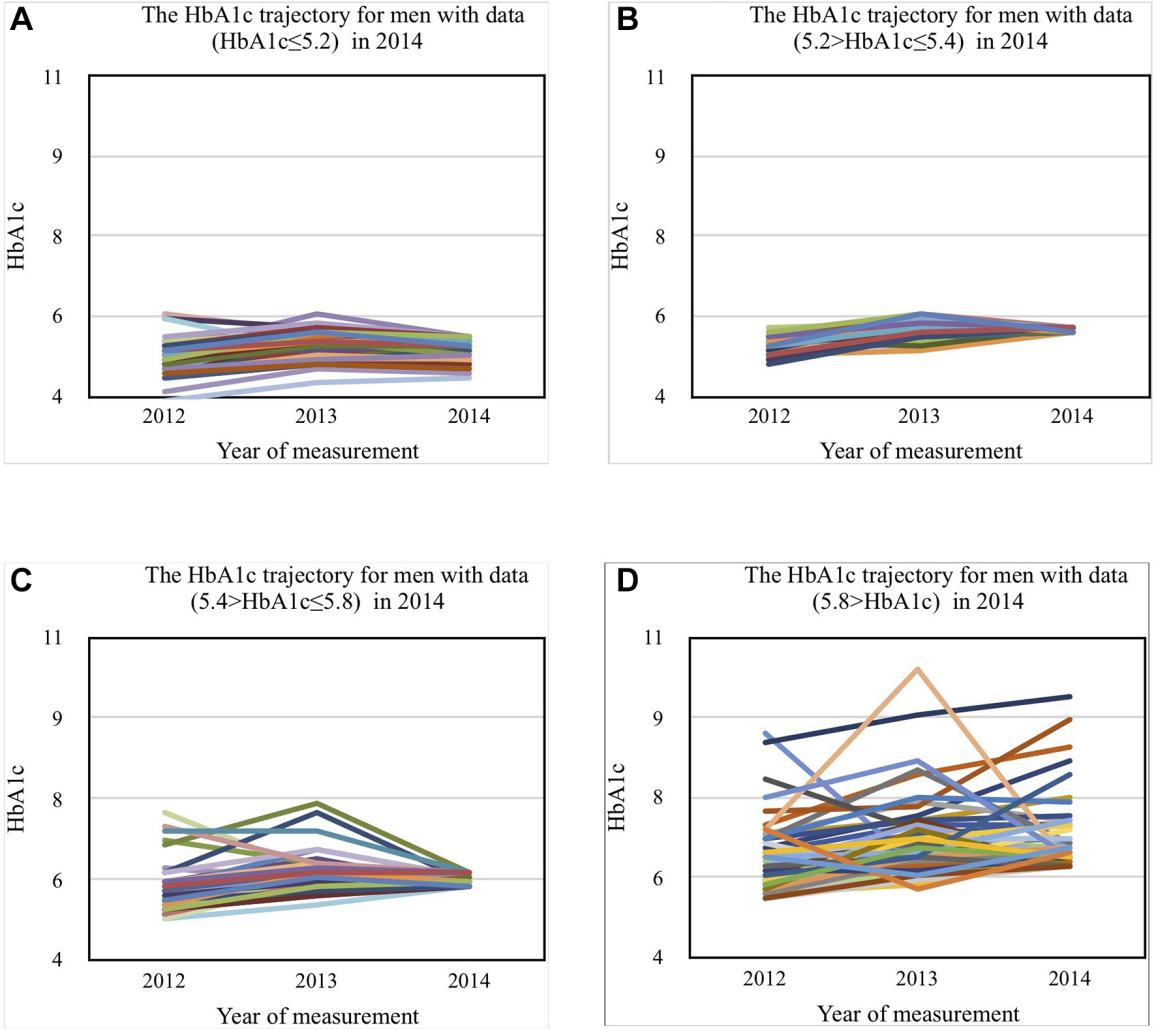
Glycated haemoglobin (HbA1c) trajectory for men.

**Figure 2:**
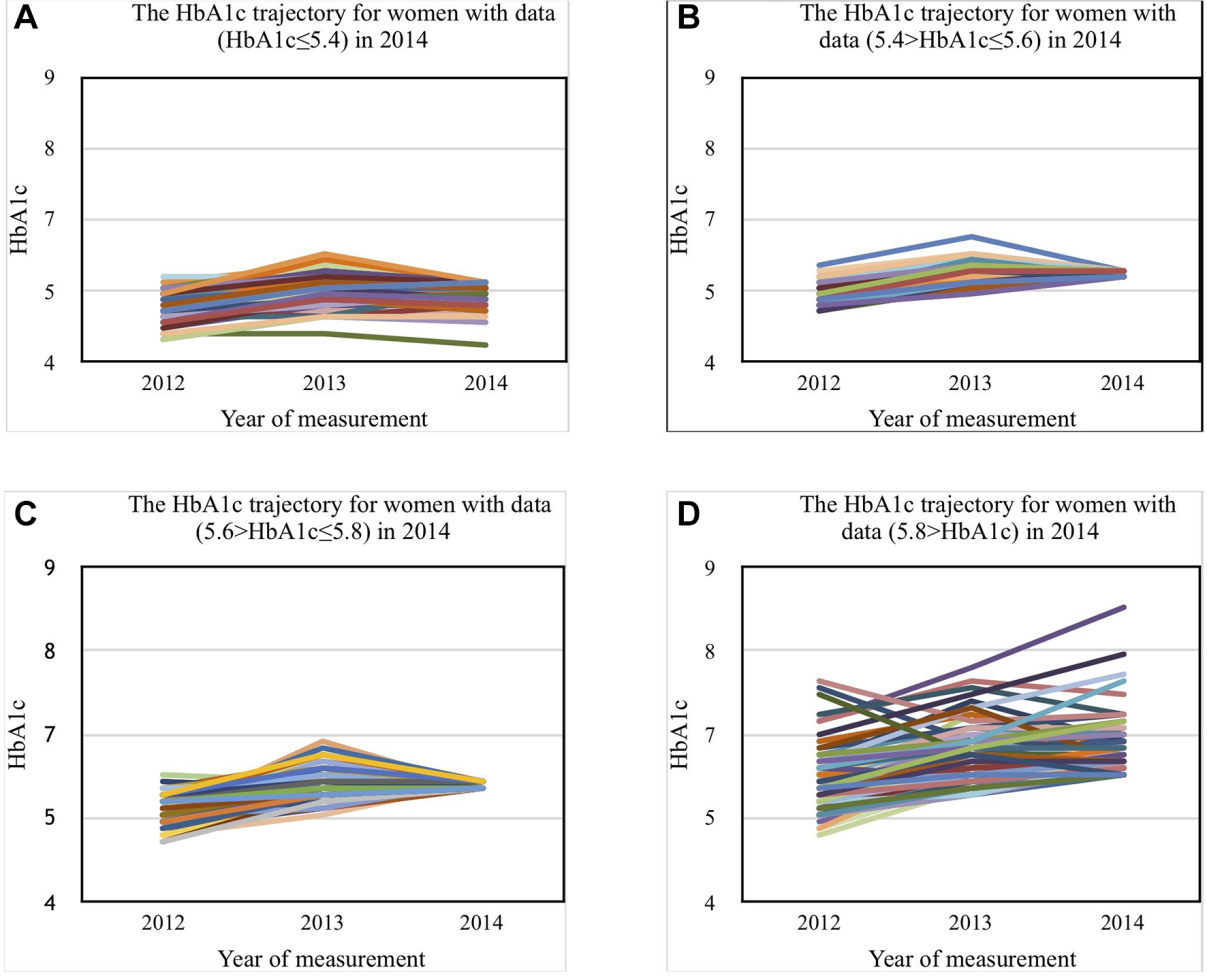
Glycated haemoglobin (HbA1c) trajectory for women.

The regression coefficients associated with HbA1c-related effects in middle-aged and elderly people are shown in table 3. Although the HbA1c value tended to rise over time in these groups, the tendency to increase in both men (figure 1) and women (figure 2) was gradual between 2013 and 2014. The HbA1c levels in 2014 were grouped in quartiles and these trajectories were displayed as four separate figures. In the 3-year period, people with normal HbA1c levels in 2014 maintained the stable and normal trajectories whereas the trajectories of high HbA1c levels tended to increase over time. These patterns were more prominent in women than in men. However, the main result was in contrast to our expectation, as sleeping within 2 hours after consuming dinner (short interval between dinner and bedtime) was reported to have no profound effect on HbA1c levels and other MetS components. There was no remarkable long-term difference in the HbA1c level between men and women. Anthropometric and lifestyle factors that could affect HbA1c levels included BMI (p<0.001), TG (p<0.001), BP (p=0.06), regular exercise (p<0.001), smoking status (p=0.013) and alcohol consumption (p=0.010), even though the effect was small—regression coefficient (β) for smoking was 0.068 (p=0.591), alcohol consumption was 0.052 (p=0.320) and regular exercise was 0.066 (p=0.12); these were not significant in the long term.

**Table 3:** Regression coefficients describing the effects of glycated haemoglobin during middle age and old age

	Null model		Model 1		Model 2		Model 3		Model 4		Model 5	
	β	P value	β	P value	β	P value	β	P value			β	P value
Intercept	5.4074	<0.001	5.2455	<0.001	5.2663	<0.001	5.3103	<0.001	5.2685	<0.001	5.3425	<0.001
Time			0.2026	<0.001	0.193	<0.001	0.2003	<0.001	0.2127	<0.001	0.2099	<0.001
Short duration between dinner and bedtime vs >2 hours					−0.02094	0.4463						
Short duration × time					0.007436	0.6507						
Higher TG in 2012 vs normal							−0.08106	<0.001				
TG × time							0.00292	0.8061				
Higher BP in 2012 year vs normal									−0.03278	0.0633		
BP × time									−0.01396	0.1859		
Higher BMI in 2012 vs normal											−0.1259	<0.001
BMI × time											−0.00917	0.4231
Men vs women												
Sex × time												
Smoking vs non-smoking												
Smoking × time												
Alcohol consumption vs no alcohol consumption												
Alcohol consumption × time												
Regular exercise vs no exercise												
Regular exercise × time												
AIC	3268.9		1737.9		1654.8		1734.4		1745.2		1703.5	
BIC	3279.6		1759.3		1675.9		1755.8		1766.7		1725	
Residual variance	0.08885	<0.001	0.04911	<0.001	0.0492	<0.001	0.0491	<0.001	0.04907	<0.001	0.04908	<0.001

	Null model		Model 6		Model 7		Model 8		Model 9		Model 10	
	β	P value	β	P value	β	P value	β	P value	β	P value	β	P value
Intercept	5.4074	<0.001	5.2412	<0.001	5.1835	<0.001	5.2027	<0.001	5.2125	<0.001	5.3261	<0.001
Time			0.1974	<0.001	0.2106	<0.001	0.192	<0.001	0.2083	<0.001	0.2169	<0.001
Short duration between dinner and bedtime vs >2 hours											0.02728	0.3135
Short duration × time											−0.00735	0.6545
Higher TG in 2012 vs normal												
TG × time												
Higher BP in 2012 vs normal												
BP × time												
Higher BMI in 2012 vs normal											−0.1388	<0.001
BMI × time											−0.01138	0.3375
Men vs women			0.006756	0.686								
Sex × time			0.008038	0.4185								
Smoking vs non-smoking					0.06839	0.0133						
Smoking × time					−0.00878	0.5907						
Alcohol consumption vs no alcohol consumption							0.05187	0.0109				
Alcohol consumption × time							0.01176	0.3202				
Regular exercise vs no exercise									0.06625	<0.001		
Regular exercise × time									−0.01521	0.12		
AIC	3268.9		1750.7		1743.5		1648.2		1641.3		1615.3	
BIC	3279.6		1772.1		1764.9		1669.3		1662.4		1636.4	
Residual variance	0.08885	<0.001	0.04912	<0.001	0.0491	<0.001	0.04921	<0.001	0.04929	<0.001	0.04917	<0.001

	Null model		Model 11		Model 12		Model 13	
	β	P value	β	P value	β	P value	β	P value
Intercept	5.4074	<0.001	5.172	<0.001	5.1949	<0.001	5.2556	<0.001
Time			0.2067	<0.001	0.2002	<0.001	0.08654	0.0983
Short duration between dinner and bedtime vs >2 hours			0.01251	0.6513	0.009657	0.7284	0.03363	0.2105
Short duration × time			−0.00671	0.6863	−0.01002	0.5462	−0.01094	0.5071
Higher TG in 2012 vs normal							0.07234	<0.001
TG × time							−0.00784	0.5272
Higher BP in 2012 vs normal								
BP × time								
Higher BMI in 2012 vs normal							−0.1232	<0.001
BMI × time							−0.01361	0.2589
Men vs women								
Sex × time								
Smoking vs non-smoking			0.06636	0.0213				
Smoking × time			0.005869	0.7277				
Alcohol consumption vs no alcohol consumption					0.05071	0.0141		
Alcohol consumption × time					0.01283	0.2836		
Regular exercise vs no exercise							−0.06409	<0.001
Regular exercise × time							0.02414	0.0184
AIC	3268.9		1661.1		1659.5		1618.9	
BIC	3279.6		1682.2		1680.6		1640	
Residual variance	0.08885	<0.001	0.04919	<0.001	0.04921	<0.001	0.04918	<0.001

AIC, Akaike information criterion; BIC, Bayesian information criterion; BMI, body mass index; BP, blood pressure; TG, triglyceride.

In addition, after analysing BMI, TG, BP, smoking status, alcohol consumption and regular exercise as covariates at the baseline, the effect of ensuring a 2-hour interval between dinner and bedtime on the HbA1c trajectory weakened. Meanwhile, the trend increased after adjusting for those covariates.

## Discussion

The baseline health status and lifestyle habits of participants were examined in 2012 and were followed-up in 2014 to investigate the influence of 2-hour interval between dinner and sleep on HbA1c level changes. To our knowledge, the effect of the duration between dinner and bedtime on HbA1c levels has not yet been studied. According to our results, people with higher HbA1c level tended to have increasing HbA1c trajectories over time. However, the main predictor variable, that is, 2-hour interval between dinner and bedtime, did not produce a significant effect on HbA1c. BMI, TG, BP, regular exercise, and smoking and alcohol consumption habits were found to influence HbA1c changes. The regression coefficients of TG, BP and regular exercise did not produce persistent associations with the main predictor and outcome variables. Therefore, there may be factors, other than the interval between the last meal of the day and bedtime, that have more profound effects on the metabolic process. For example, dietary components such as major macronutrients and micronutrients are crucial for blood glucose modulation.^28^ Japan also has a regulatory system for healthy food and a nutritional policy to maintain the beneficial effects of various products.^29 30^ Therefore, the traditional meal culture and nutritional components of meals in the country may not pose problems.^7^ Nevertheless, the assessment of dietary patterns is beyond the scope of our study and these could not be interpreted directly from the results of our study. Similarly, elderly people often have problems such as social isolation, economic problems^31^ and impaired oral status^32^ that could decrease their ability to eat. In our study, two-thirds of the participants were aged over 65 years and retired; they may have had a poorer nutritional status and been more vulnerable to having reduced HbA1c levels than the younger participants.

Meal timing is an important factor in the maintenance of a stable metabolic process.^25^ Among Americans, the density of food in dinner is significantly higher than that in any other meal. Eating at night, eating snacks between meals, and unusual eating patterns are related to a lower intake of fruits and vegetables and a higher intake of sweets and fat.^33^ Therefore, food contents, particularly those of meals consumed later in the day, also play an important role in stable metabolic process maintenance. These factors could have a more profound effect on age groups comprising working people and younger people as they are highly likely to engage in social eating and have inadequate sleeping habits. A Japanese cross-sectional study showed that eating dinner late at night was associated with hyperglycaemia in the general population. Interventions for such unhealthy behaviour should be a priority for the prevention of cardio-metabolic complications.^21^ However, the short interval between dinner and bedtime did not produce significant HbA1c level changes in our study. As we could not conclude the definite time interval required between dinner and sleep, it was difficult to define ‘late dinner’. Regarding sleeping status, a U-shape association was observed between sleep duration and HbA1c level.^22^ Extremely long and short sleeping hours were related to increased HbA1c levels even in the short term. Similarly, higher food intakes closer to bedtime had a negative effect on sleep quality.^34^

Although the presence of a short interval between dinner and bedtime did not significantly influence the HbA1c levels in our study, this result could not be generalised because of the cultural effect of the healthy food contents and eating style in Japan. Ensuring healthy portions and paying attention to the nutrient contents of meals could be more useful in maintaining normal glucose metabolism than ensuring a particular interval between the last meal of the day and bedtime. Consequently, the influence of smoking and alcohol consumption habits must be considered. In our study, smoking and alcohol consumption caused significant changes in the HbA1c levels. A Japanese study found that smoking and alcohol consumption were related to an increased MetS development rate.^35^ Alcohol consumption was not consistently related to increasing HbA1c levels,^36 37^ whereas smoking has always been shown to have an unfavourable effect on the same.^38^ In Japan, the prevalence rates of smoking are 57.0% and 16.6%, respectively, among men and women^39^; the corresponding values for alcohol consumption are 36.9% and 12.0%, respectively.^40^ People have a general tolerant attitude toward alcohol consumption. These social factors should be considered in developing measures for the maintenance of a stable long-term metabolic process.

Our study has some limitations. First, analyses of the categorical data on the lifestyle variables could not provide sufficient power for the achievement of statistical significance compared with those of continuous data in the interval between dinner and bedtime and the length of sleep. Second, it would have been ideal to collect data on the definite time interval between dinner and bedtime as it would have provided more accurate information on the healthy hours for eating and sleeping, and their association with normal HbA1c levels. Additionally, due to the absence of data on dietary components, the prediction of HbA1c level changes by meal content could not be supported. Dietary components should be considered as important determinants in future studies. Third, as our sample comprised middle-aged and elderly people, the generalisability of the results is limited, and the results cannot be applied to age groups including working people. There may be lifestyle-related differences between these groups. Consequently, the effect of the time interval between eating dinner and sleep on HbA1c levels could be different in the age group comprising working people. Despite these limitations, the use of 3-year data in the current study was useful in identifying HbA1c change trajectories over time. Moreover, we were able to examine the effect of lifestyle variables on increasing HbA1c levels over time; this helped in developing an EBP for preventive measures against lifestyle-related diseases.

## Conclusion

Contrary to general belief, ensuring a short interval between the last meal of the day and bedtime did not significantly affect HbA1c levels. More attention should be paid to healthy portions and food components, getting adequate sleep, and avoiding smoking, alcohol consumption and overweight development, as these variables had a more profound influence on the metabolic process.
